# Usefulness of the endoscopic surgical skill qualification system in laparoscopic colorectal surgery: short-term outcomes: a single-center and retrospective analysis

**DOI:** 10.1186/s12893-019-0528-2

**Published:** 2019-07-11

**Authors:** Shota Aoyama, Yuji Inoue, Takeshi Ohki, Michio Itabashi, Masakazu Yamamoto

**Affiliations:** Department of Gastroenterological Surgery, Tokyo Women’s Medical University, 8-1, Kawada-cho, Shinjuku-ku, Tokyo, 162-8666 Japan

**Keywords:** Endoscopic surgical skill qualification system, Qualified surgeon, Laparoscopic surgery, Colorectal cancer, Short-term outcomes, Propensity score matching

## Abstract

**Background:**

The use of laparoscopic surgery has become widespread, and many surgeons are striving to acquire the necessary techniques for it. The Endoscopic Surgical Skill Qualification System (ESSQS), established by the Japan Society for Endoscopic Surgery, serves to maintain and improve the quality of laparoscopic surgery in Japan. In this study, we aimed to determine whether ESSQS certification is useful in maintaining and improving the quality of surgical techniques and in standardization of laparoscopic surgery in Japan.

**Methods:**

This retrospective study used data from the Institute for Integrated Medical Sciences, Tokyo Women’s Medical University**,** Japan. From January 2016 to October 2017, 241 patients with colorectal cancer underwent laparoscopic surgery. Of them, 220 patients were selected and divided into two groups on the basis of surgery performed by an ESSQS-qualified surgeon (QS group) (*n* = 170) and a non-ESSQS-QS (NQS) (*n* = 50). We compared the short-term results in the two groups and examined those before and after propensity score matching (PSM).

**Results:**

Mean operation time was longer in the NQS group than in the QS group. Furthermore, mean blood loss was significantly less in the QS group. These were similar before and after PSM. The rate of conversion to open surgery was significantly higher in the NQS group before PSM. However, the rate of postoperative complications was not different between the two groups.

**Conclusions:**

A laparoscopic procedure performed by ESSQS-QS often leads to good short-term outcomes. Thus, the ESSQS system works and is potentially useful in maintaining and improving the quality of surgical techniques and in standardization of laparoscopic surgery in Japan.

## Background

The use of laparoscopic surgery has become widespread because this technique can be easily adapted to various organs and disease treatments. In colorectal surgery, using a laparoscopic rather than open approach generally leads to faster recovery, reduced duration of postoperative ileus, lower wound infection rates, shorter hospital stay, reduced postoperative pain, and earlier tolerance of a regular diet [1, 2]. However, laparoscopic surgical techniques are difficult to master, and a laparoscopic approach is associated with an increased operation time compared with an open approach [1, 2]. In an attempt to standardize laparoscopic surgery in Japan, the Endoscopic Surgical Skill Qualification System (ESSQS), established by the Japan Society for Endoscopic Surgery (JSES), serves to maintain and improve the quality of surgical technique and to standardize laparoscopic surgery [3–5]. Applicants who want to have the privileges of endoscopic surgeons are required to submit certain documents, including a letter certifying 2 years of uninterrupted endoscopic surgical practice after completion of all formal training, a certificate of membership of the JSES, and the special board of the Japan Society of Surgery, certificates of attendance of meetings and seminars held under the auspices of the JSES, a bibliography showing papers presented at the meetings or papers published in the authorized journals of the JSES, in addition to a list of endoscopic surgeries the applicant has performed by himself or herself over the last 3 years, together with an unedited Video, showing the surgery carried out by his or her own effort, and suturing and knotting techniques the applicant performed by him or herself. They are all screened and evaluated very seriously by committee members elected from individual Committees in order to make a final decision. For video evaluation, two judges, elected from the individual society, review the video using a score sheet, with detailed checking points and mark allocation. Checking points are divided into 2 parts consisting of: “common criteria” for basic endoscopic techniques commonly used for all procedures, and “organ-specific criteria” for special endoscopic surgical techniques for individual organs. The allotted marks for each criterion are 60 and 40 points respectively. The evaluation is focused on surgical techniques and camera work and a total score of 70 points is designated as the pass mark. The number of certificate holders certified in the field of Gastroenterological Surgery during the period from 2003 to 2012 is around 1000, with an average success rate of around 50%. The main reason for the low success rate is attributable to their mainly immature techniques including careless handling of organs with inadequate instruments, or an inadequate operative field and a lack of communication among operators. [4]

This certification system is original and unmatched worldwide, and it is expected to be extremely useful for improving surgical outcomes and reducing complications. However, studies proving the usefulness of ESSQS have not been published since 2004. In this study, we aimed to determine whether ESSQS is useful in maintaining and improving the quality of surgical techniques and in the standardization of laparoscopic surgery in Japan.

## Materials and methods

We focused on laparoscopic colorectal surgery and limited our study only to patients who underwent colorectal cancer surgery and in whom the pathology was diagnosed using postoperative pathological specimens, excluding those with autoimmune diseases, benign diseases, and malignant lymphomas. In this retrospective case-controlled study, we used data from the Institute for Integrated Medical Sciences, Tokyo Women’s Medical University**,** Japan.

**Fig. 1:**
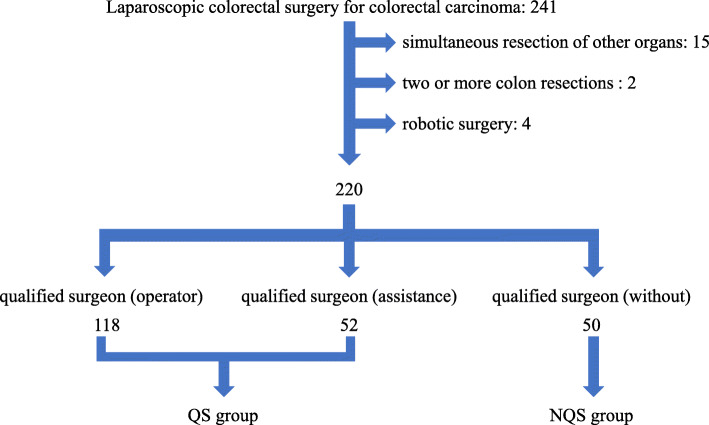
Two hundred forty-one patients with colorectal cancer underwent laparoscopic surgery. We excluded patients with the following: simultaneous resection of the other organs (*n* = 15), two or more colon resections (*n* = 2), and robot-assisted surgery (*n* = 4). We divided remaining patients into two groups: QS group (*n* = 170) and NQS group (*n* = 50). Surgical team in QS group included a lead surgeon, assistant, or camera operator. In the QS group, 118 surgeries were performed with QS, and there was either an assistant or camera operator in 52 surgeries. For the NQS group, no ESSQS-qualified surgeons participated

From January 2016 to October 2017, 241 patients with colorectal cancer underwent laparoscopic surgery. Excluding patients with simultaneous resection of the other organs (*n* = 15), two or more colon resections in the same operation (*n* = 2), and robot-assisted surgery (*n* = 4), the remaining 220 patients were included. Patients were divided into two groups on the basis of surgery performed by an ESSQS-qualified surgeon (QS group) (*n* = 170) and a non-ESSQS-QS (NQS) (*n* = 50). The surgical team in the QS group generally included an ESSQS-qualified lead surgeon, a laparoscopic surgical assistant, or a camera operator. In the QS group, 118 surgeries were performed with QS; of them, 52 surgeries were performed with NQS in presence of a laparoscopic surgical assistant or camera operator with QS (Fig. 1). In the NQS group, ESSQS-QS did not participate in any of the surgeries.

At our facility, we conduct group medical examination based on the organ, and there are 10 surgeons in the colorectal group. They all possess more than 5 years of surgical experience, and three of them are ESSQS qualified. In QS surgeon group, One surgeon with more than 15 years of experience, one with more than 20 years and one with more than 25 years. On the other hand, in another group, five surgeons have experience as a surgeon for more than 10 years, in addition, one more than 20 years and one had more than 25 years’ experience. Generally, laparoscopic procedures included three physicians from our team: lead surgeon, assistant, and camera operator. All surgical procedures and roles of participating physicians were decided at preoperative team staff conferences, and almost every surgery proceeded as planned. The extent of lymph node dissection was decided by the lead surgeon before the surgery. However, our result of this time derived the extent of dissection from the pathological result after the operation.

We retrospectively analyzed the following short-term outcomes after laparoscopic surgeries: operation time (min), blood loss (ml), conversion to open surgery, lymph node dissection level, number of lymph nodes harvested, postoperative complications, and postoperative stay in the hospital (day). Conversion to open surgery was intraoperatively judged by the lead surgeon. Patient management during the perioperative period was similar in all cases except for minor variations in perioperative antibiotics because of group medical examination. Postoperative complications were evaluated according to the Clavien–Dindo classification [6]. All enrolled patients gave their informed consent. This study was approved by the institutional review board of the Tokyo Women’s Medical University Hospital.

### Statistical analysis

We used JMP Pro 11 software (SAS Institute, Cary, NC, USA) for all statistical analyses. All quantitative variables were reported as means. Continuous variables for the two groups were checked for normality of distribution using one-sample Kolmogorov–Smirnov test and compared using analysis of variance (Student’s *t*-test or Mann–Whitney U test). Binomial and categorical data were evaluated by cross-linked tables using Pearson’s v^2^ or two-tailed Fisher’s exact tests. Univariate analysis (non-parametric Wilcoxon two-sample test for continuous variables and Χ^2^ test for categorical variables) was performed for both groups. To identify independent risk factors for short-term outcomes using multivariate analysis, all significant variables evaluated on univariate analysis were integrated into multiple logistic regression and multiple regression analyses. *P* < 0.05 was considered significant (Table 1).

**Table 1:** Univariate analysis and multivariate analysis of bleeding, operation time, and conversion

Bleeding	Univariate	Multivariate	operation time	Univariate	Multivariate	Conversion	Univariate	Multivariate
Age	0.176	0.08474		0.5061	0.53396		0.2371	0.00001*
Gender	0.0602	0.13326		0.0204*	0.07094		0.4712	0.00001*
ASA	0.6456	0.82847		0.1773	0.1221		0.1401	0.00001*
BMI	0.2677	0.3456		0.0773	0.02911*		0.4072	0.99985
Location	0.9927	0.48422		0.0001*	0.02309*		0.1272	0.99512
Operation	0.1728	0.07998		0.0001*	0.00001*		0.9072	0.00243*
Anastomosis	0.809	0.27839		0.0001*	0.00283*		0.0897	0.00001*
History of abdominal surgery	0.8172	0.95322		0.0844	0.05482		0.532	0.73795
NQS	0.0488*	0.08287		0.0001*	0.00001*		0.0005*	0.00001*

*P < 0.05

### Propensity score matching

We used PSM to minimize the differences in baseline characteristics between the QS and NQS groups. The following variables were included to establish the model: age, gender, body mass index (BMI), the American Society of Anesthesiologists (ASA) score, history of abdominal surgery, stage, anastomosis, tumor size, tumor location, operative procedure. After PS generation, patients in the QS and NQS groups underwent 1:1 nearest available matching of the logit of the propensity score with a caliper width of 0.20 of the standard deviation of the score. Patients who did not meet the matching criteria were excluded.

## Results

### Patient demographic characteristics

We were able to assign 220 included patients to either of the two groups (Fig. 1). Table 2 compares the characteristics of the patients in two groups. There was no difference between the QS and NQS groups regarding age (68.49 vs 66.30 years, *P* = 0.2273), gender (*P* = 0.4001), and BMI (22.21 vs 22.01 kg/m^2^, *P* = 0.694). Preoperative physical status was scored using ASA classification, and all the patients were ASA class I, II, or III, with no difference between the groups (*P* = 0.292). To compare the possibility of adhesion during the surgery, surgical history of the abdomen was compared, but no significant differences were found (32.94% vs 42%, *P* = 0.2378); furthermore, there was no difference in the mean size of tumor in the two groups (35.51 vs 37.34 mm, *P* = 0.5781). Various surgical procedures were performed; laparoscopic-assisted ascending colon resection (LACAR) and laparoscopic-assisted Hartmann’s operation (LA Hartmann’s operation) were only performed in the QS group; however, there was no significant difference between these two surgical procedures (*P* = 0.830). Regarding the method of anastomosis, there was a significant difference (*P* = 0.0002) between the two groups; functional end-to-end anastomosis (FEEA) was more frequently used in the QS group (56.47% vs 36%, *P* = 0.0109), whereas handsewn anastomosis was more frequently performed in the NQS group (0.59% vs 12%, *P* = 0.001), and Albert-Lembert suture was performed in almost all the handsewn anastomoses. The 1:1 PS-matched cohort comprised 43 patients from the QS group and 43 from the NQS group. The differences in patient characteristics between the QS and NQS groups in the original analysis were alleviated after PSM (Table 2). No significant difference was observed in any item.

**Table 2:** Patient characteristics before and after PSM

Before PSM	QS *n*?=?170	NQS *n*?=?50	*p* value	After PSM	QS *n*?=?43	NQS *n*?=?43	*p* value
Mean age (years)	68.49	66.3	0.2273		65.65	65.19	0.8589
Gender female	44.71% (*n*?=?76)	38%(*n*?=?19)	0.4001		39.53% (*n*?=?17)	39.53% (*n*?=?17)	1
Mean BMI (Kg/m^2^)	22.21	22.01	0.694		21.65	22.37	0.293
ASA			0.292				0.8204
Class I	7.65% (*n*?=?13)	14% (*n*?=?7)	0.1696		11.63% (*n*?=?5)	16.28% (*n*?=?7)	0.5337
Class II	72.35% (*n*?=?123)	72% (*n*?=?36)	0.9609		76.74% (*n*?=?33)	72.09% (*n*?=?31)	0.6211
Class III	20% (*n*?=?34)	14% (*n*?=?7)	0.3382		11.63% (*n*?=?5)	11.63% (*n*?=?5)	1
History of abdominal surgery +	32.94% (*n*?=?56)	42% (*n*?=?21)	0.2378		32.56% (*n*?=?14)	34.88% (*n*?=?15)	0.8196
Stage			0.6068				0.5808
0	3.53% (*n*?=?6)	4% (*n*?=?2)	0.8758		4.65% (*n*?=?2)	4.65% (*n*?=?2)	1
I	28.24 (*n*?=?48)	30% (*n*?=?15)	0.8083		37.21% (*n*?=?16)	25.58% (*n*?=?11)	0.2453
II	31.18% (*n*?=?53)	22% (*n*?=?11)	0.2092		18.6% (*n*?=?8)	20.93% (*n*?=?9)	0.7866
IIIa	16.47% (*n*?=?28)	26% (*n*?=?13)	0.1282		13.95% (*n*?=?6)	27.91% (*n*?=?12)	0.1117
IIIb	9.41% (*n*?=?16)	6% (n?=?3)	0.4571		4.65% (*n*?=?2)	6.98% (*n*?=?3)	0.7142
IV	11.18% (*n*?=?19)	12% (*n*?=?6)	0.8719		20.93% (*n*?=?9)	13.95% (*n*?=?6)	0.3939
Anastomosis			0.0002*				0.7737
FEEA	56.47% (*n*?=?96)	36% (*n*?=?18)	0.0109*		44.19% (*n*?=?19)	41.86% (*n*?=?18)	0.8276
DST	40.59% (*n*?=?69)	50% (*n*?=?25)	0.237		51.16% (*n*?=?22)	55.81% (*n*?=?24)	0.6656
handsewn	0.59% (*n*?=?1)	12% (*n*?=?1)	0.0001*		2.33% (*n*?=?1)	0% (*n*?=?0)	0.3145
none	2.35% (*n*?=?4)	2% (*n*?=?1)	0.883		2.33% (*n*?=?1)	2.33% (*n*?=?1)	1
Tumor size (mm)	35.51	37.34	0.5781		36.28	36.79	0.9043
Location			0.0945				0.8245
colon	77.65% (*n*?=?132)	66% (*n*?=?33)	0.0945		60.47% (*n*?=?26)	62.79% (*n*?=?27)	0.8245
rectum	22.35% (*n*?=?38)	34% (*n*?=?17)	0.0945		39.53% (*n*?=?17)	37.21% (*n*?=?16)	0.8245
Operation			0.83				0.712
LAAPR	1.18% (*n*?=?2)	2% (*n*?=?1)	0.6589		2.33% (*n*?=?1)	2.33% (*n*?=?1)	1
LAAR	11.76% (*n*?=?20)	18% (*n*?=?9)	0.2519		20.93% (*n*?=?9)	20.93% (*n*?=?9)	1
LACAR	1.18% (*n*?=?2)	0% (*n*?=?0)	0.441		2.33% (*n*?=?1)	0% (*n*?=?0)	0.3145
LACDR	1.18% (*n*?=?2)	2% (*n*?=?1)	0.6589		2.33% (*n*?=?1)	0% (*n*?=?0)	0.3145
LACSR	22.35% (*n*?=?38)	20% (*n*?=?10)	0.7233		11.63% (*n*?=?5)	16.28% (*n*?=?7)	0.5337
LACTR	7.65% (*n*?=?13)	4% (*n*?=?2)	0.3685		9.3% (*n*?=?4)	2.33% (*n*?=?1)	0.1668
LA Hartmann’ operation	1.18% (*n*?=?2)	0% (*n*?=?0)	0.441		0% (*n*?=?0)	0% (*n*?=?0)	1
LAICR	12.35% (*n*?=?21)	8% (*n*?=?4)	0.3939		11.63% (*n*?=?5)	9.3% (*n*?=?4)	0.7246
LALAR	12.94% (*n*?=?22)	18% (*n*?=?9)	0.3661		23.26% (*n*?=?10)	18.6% (*n*?=?8)	0.596
LALt.hemi.CR	6.47% (*n*?=?11)	10% (*n*?=?5)	0.5936		4.65% (*n*?=?2)	9.3% (*n*?=?4)	0.3972
LARt.hemi.CR	21.76% (*n*?=?37)	18% (*n*?=?9)	0.4571		11.63% (*n*?=?5)	20.93% (*n*?=?9)	0.2427

*P < 0.05

### Short-term outcome

Table 3 shows short-term outcomes in the two groups before and after PSM. Compared with the QS group, mean operation time was significantly longer in the NQS group (213.4 min vs 291.7 min, *P* = 0.0001, 221.6 min vs 304.6 min, *P* = 0.007). Blood loss was minimal in both groups; however, mean bleeding amount was significantly less in the QS group (25.52 ml vs 45.54 ml, *P* = 0.0488, 17.47 ml vs 48.6 ml, *P* = 0.0436). Lymph node dissection level was no significant differences were observed after PSM (*P* = 0.3833). Regarding the number of harvested lymph nodes, the number of exploited lymph nodes was higher in the QS group (20.19 vs 12.42, *P* = 0.0001, 18.74 vs 12.44, *P* = 0.003). Conversion to open surgery occurred in four cases, all in the NQS group (0% vs 8%, *P* = 0.0002) before PSM. However, after PSM, no significant differences were observed (0% vs 6.98%, *P* = 0.0779). Regarding complications, no significant difference was observed between the groups (25.29% vs 28%, *P* = 0.7011, 27.91% vs 25.58%, *P* = 0.8075); furthermore, the Clavien–Dindo classification showed no difference between the groups. One case in each group required surgery because of a major anastomotic leak. There was no difference between the groups regarding postoperative hospital stay (13.15 days vs 13.78 days, *P* = 0.6057, 12.56 days vs 13.63 days, *P* = 0.3618). On multivariate analysis, surgical time in the QS group was detected as a significant factor (*P* = 0.0001), including the gender and surgical method. No significant factor could be detected by multivariate analysis regarding bleeding volume. Regarding the rate of conversion to an open procedure, being in the NQS group became a factor with a significant difference (*P* = 0.0003).

**Table 3:** Operative and postoperative results before and after PSM

Before PSM	QS *n*?=?170	NQS *n*?=?50	*p* value	After PSM	QS *n*?=?43	NQS *n*?=?43	*p* value
Operation time (min)	213.4	291.7	0.0001*		221.6	304.6	0.0007*
Blood loss (ml)	25.52	45.54	0.0488*		17.47	48.6	0.0436*
Conversion	0% (*n*?=?0)	8% (*n*?=?4)	0.0002*		0% (*n*?=?0)	6.98% (*n*?=?3)	0.0779
Harvested lymph nodes	20.19	12.42	0.0001*		18.74	12.44	0.003*
Complications	25.29% (*n*?=?43)	28% (*n*?=?14)	0.7011		27.91% (*n*?=?12)	25.58% (*n*?=?11)	0.8075
Clavien–Dindo							
I	15.29% (*n*?=?26)	18% (*n*?=?9)	0.6456		18.6% (*n*?=?8)	16.28% (*n*?=?7)	0.7763
II	7.65% (*n*?=?13)	6% (*n*?=?3)	0.6934		4.65% (*n*?=?2)	6.98% (*n*?=?3)	0.6449
IIIa	1.76% (*n*?=?3)	2% (*n*?=?1)	0.9128		4.65% (*n*?=?2)	2.33% (*n*?=?1)	0.5567
IIIb	0.58% (*n*?=?1)	2% (*n*?=?1)	0.3552		0% (*n*?=?0)	0% (*n*?=?0)	1
Hospital stay after surgery (day)	13.15	13.78	0.6057		12.56	13.63	0.3618
Lymph node dissection level			0.0008*				0.3833
D0	3.53% (*n*?=?6)	6% (*n*?=?3)	0.4382		2.33% (*n*?=?1)	6.98% (*n*?=?3)	0.3068
D1	2.35% (*n*?=?4)	4% (*n*?=?2)	0.5296		0% (*n*?=?0)	2.33% (*n*?=?1)	0.3145
D2	10% (*n*?=?17)	32% (*n*?=?16)	0.0001*		18.6% (*n*?=?8)	25.58% (*n*?=?11)	0.4355
D3	82.35% (*n*?=?143)	58% (*n*?=?29)	0.0001*		79.07% (*n*?=?34)	65.12% (*n*?=?28)	0.1492

*P < 0.05

## Discussion

Laparoscopic surgery is being performed worldwide at many facilities. While it has many positive aspects for patients, it is technically challenging for the surgeon and surgical team [1, 2]. There is a push to standardize laparoscopic surgery, and ESSQS serves to maintain and improve the quality of surgical technique and the standardization of laparoscopic surgery in Japan [3–5]. This system is unique to Japan and unmatched elsewhere in the world. There are few published articles related to ESSQS, so it is very difficult to find proof of its effectiveness in the literature [6–11]. Through our retrospective single-facility study, we showed the usefulness of ESSQS for the first time.

There were no differences regarding patient background in our study, but there was a difference in the proportion of intestinal anastomosis during surgery. At our facility, the choice of anastomosis during surgery is left to the lead surgeon. There are cases where the intestinal tract length is insufficient and stapled anastomosis is difficult, but there is a possibility that this is determined by a surgeon who is not proficient in handsewn anastomosis. The possibility of difference in the anastomosis method influencing the operation time and bleeding amount in this study cannot be denied. Regarding anastomotic leakage, Choy concluded that stapled anastomosis results in less leakage than handsewn anastomosis [12–17]. In addition, data on short surgical time is recognized [14, 18]. Regarding bleeding, we did not acknowledge the paper which recognizes a big difference in the range to be examined [15, 16].

The results of our study show that the surgical time and bleeding amount were significantly lower in the QS group. On comparing with the JCOG 0404 study on bleeding volume and surgery time, the QS group showed nearly comparable results, but the NQS group showed longer operation time and greater bleeding amount [19]. This indicates that ESSQS-QS may be able to control bleeding more accurately, perform the standardized procedure faster, and instruct other physicians in the technique. Regarding operation time, multivariate analysis showed ESSQS qualification to be a risk factor along with gender, tumor site, operation method, and anastomosis method. However, regarding the amount of bleeding, ESSQS qualification was not found to be a risk factor in multivariate analysis. Because laparoscopic surgery generally involves a small amount of bleeding, there is a possibility that it is difficult to detect as a significant difference. However, to eliminate these differences, we decided to further modify with PSM and compare the two groups. After PSM, a difference was observed in the amount of bleeding and operation time. However, in our study, it was difficult to compare assistants and cameras separately for surgical assistants. The camera handling by an unexperienced surgeon is one of the major factors in time loosing even when the lead surgeon is experienced in laparoscopic surgery. Also, the same can be said for the assistant. Of course, it could also cause bleeding.

Conversion to open surgery occurred in four cases in the NQS group because of adhesion. Although it is difficult to evaluate the rate of conversion to an open approach, the results of a multicenter randomized controlled trial showed that conversion to open surgery has increased in laparoscopic colorectal resection cases in patients with physical status ASA III and above, hemi-right or left colon resection, sigmoidectomy, low anterior resection, or abdominoperineal resection [19–25]. However, a recent study reported that there was no correlation between ASA and BMI regarding laparotomy conversion rate [26, 27]. Conversely, a study reported that the rate of conversion increased in cases with BMI ≥ 27.5 kg /m^2^, with the rate of conversion being as high as ≥20%. It was also noted that many surgeons did not reach the learning curve for ESSQS qualification, so definite prediction it is difficult to judge as a factor [23]. In our study, the lead surgeon determined conversion to an open approach. There is a high possibility that surgery can be completed without laparotomy by more experienced surgeons; however, conversion is by no means a surgical error if it is necessary for patient safety. We believe that safely performing a surgery depends on surgeons’ skills as well. However, these results could not be confirmed by the modified PSM comparison because our study changed the number of patients who converted into laparotomy surgery, further accumulation of cases is necessary in the future.

Regarding lymph node dissection and the number of harvested lymph nodes, the QS group was more widely dissected and many lymph nodes could be collected. This results of the extent of dissection are indicated by postoperative pathological results. The extent of lymph node dissection was discussed before surgery, surgery was performed accordingly. However, in this study, the final lymph node dissection extent was defined by the presence of lymph nodes, with or without metastasis. This result also reflects that ESSQS-QS can perform more precise dissections and is also familiar with D3 cases. However, because troubleshooting such as response to bleeding may be more effective with ESSQS- QS, dissection can be more precise in the QS group. Also, with more accurate technique, more accurate dissection might have been possible in the QS group. The number of lymph node dissections in our study was not significantly different from those reported in other studies [28]. However, these results also showed no difference after PSM. Considering this, it is highly likely that the number of lymph node dissection levels do not lead to a difference in the two groups. To lead out these conclusions, further accumulation of cases and improvements in study methods are required.

There was no significant difference in postoperative complications and hospital stay between the QS and NQS groups, and in each group, surgery became necessary in only one case due to a major leakage. Both groups show a good postoperative course. Even with the JCOG0404 study, the rate of complications was reasonable [18]. There was no difference between the groups regarding postoperative hospital days. As postoperative management of patients at our facility is performed by a team, there was little difference between the groups regarding the timing of discharge and judgment of complications. Also, postoperative management is unified. Thus, there is a possibility that this result may have strong evidence.

Based on the abovementioned results, the ESSQS certification process for colorectal surgical technique is accurately performed by the association. In a study similar to ours, Nijhof et al. compared experts and residents for laparoscopic colorectal surgical skills and obtained results different from ours. This may indicate the difference between an expert surgeon and ESSQS- QS [29]. On the other hand, another evaluation of technique for certification is a surgeon meeting the predetermined criteria as evaluated by another, more experienced surgeon. Also, in this study, we compared, as a subsidiary, a group of interventions between ESSQS-QS who assisted the non-ESSQS-QS and non-ESSQS-QS. As seen in Tables 4 and 5, before PSM surgery time, laparotomy conversion rate, and lymph node dissection showed significantly better results for the interventions by ESSQS- QS. Also, after PSM showed significantly better results in operation time and harvested lymph nodes. This result is similar to those reported in the literature and is one of the indicators of the quality of technical certification [30].

**Table 4:** Patient characteristics in QSA and NQS groups before and after PSM

Before PSM	QSA *n* = 52	NQS *n* = 50	*p* value	After PSM	QSA *n* = 33	NQS *n* = 33	*p* value
Mean age (years)	68.21	66.3	0.4076		67.76	67.24	0.8625
Gender Female	42.31% (*n* = 22)	38% (*n* = 19)	0.6573		42.42% (*n* = 19)	39.39% (*n* = 20)	0.8023
Mean BMI (Kg/m^2^)	22.1	22.01	0.8865		22.94	22.36	0.4978
ASA			0.2356				0.8669
Class I	5.77% (*n* = 3)	14% (*n* = 7)	0.1623		9.09% (*n* = 3)	12.12% (*n* = 4)	0.6893
Class II	71.15% (*n* = 37)	72% (*n* = 36)	0.9245		78.79% (*n* = 26)	78.79% (*n* = 26)	1
Class III	23.08 (*n* = 12)	14% (*n* = 7)	0.2392		12.12% (*n* = 4)	9.09% (*n* = 3)	0.6893
History of abdominal surgery +	34.62% (*n* = 18)	42% (*n* = 21)	0.443		42.42% (*n* = 14)	33.33% (*n* = 11)	0.4465
Stage			0.6546				0.9823
0	7.69% (*n* = 4)	4% (*n* = 2)	0.4282		6.06% (*n* = 2)	6.06% (*n* = 2)	1
I	34.62% (*n* = 18)	30% (*n* = 15)	0.6184		27.27% (*n* = 9)	30.3% (*n* = 10)	0.7857
II	25% (*n* = 13)	22% (*n* = 11)	0.721		27.27% (*n* = 9)	21.21% (*n* = 7)	0.5657
IIIa	17.31% (*n* = 9)	26% (*n* = 13)	0.286		24.24% (*n* = 8)	21.21% (*n* = 7)	0.769
IIIb	9.62% (*n* = 5)	6% (*n* = 3)	0.561		6.06% (*n* = 2)	9.09% (*n* = 3)	0.6121
IV	5.77% (*n* = 3)	12% (*n* = 6)	0.2674		9.09% (*n* = 3)	12.12% (*n* = 4)	0.6893
Anastomosis			0.0096*				0.6223
FEEA	61.54% (*n* = 32)	36% (*n* = 18)	0.0099*		51.52% (*n* = 17)	45.45% (*n* = 15)	0.6223
DST	38.46% (*n* = 20)	50% (*n* = 25)	0.2407		48.48% (*n* = 16)	54.55% (*n* = 18)	0.6223
handsewn	0% (*n* = 0)	12% (*n* = 6)	0.01*		0% (*n* = 0)	0% (*n* = 0)	1
none	0% (*n* = 0)	2% (*n* = 1)	0.3054		0% (*n* = 0)	0% (*n* = 0)	1
Tumor size (mm)	32.84	37.34	0.2478		35.85	35.36	0.918
Location			0.0289*				0.2689
colon	84.62% (*n* = 44)	66% (*n* = 33)	0.0289*		78.79% (*n* = 26)	66.67% (*n* = 22)	0.2689
rectum	15.38% (*n* = 8)	34% (*n* = 17)	0.0289*		21.21% (*n* = 7)	33.33% (*n* = 11)	0.2689
Operation			0.1473				0.8838
LAAPR	0% (*n* = 0)	2% (*n* = 1)	0.3054		0% (*n* = 0)	0% (*n* = 0)	1
LAAR	15.38% (*n* = 8)	18% (*n* = 9)	0.7231		18.18% (*n* = 6)	24.24% (*n* = 8)	0.547
LACAR	0% (*n* = 0)	0% (*n* = 0)	1		0% (*n* = 0)	0% (*n* = 0)	1
LACDR	0% (*n* = 0)	2% (*n* = 1)	0.3054		0% (*n* = 0)	0% (*n* = 0)	1
LACSR	26.92% (*n* = 14)	20% (*n* = 10)	0.4099		24.24% (*n* = 8)	18.18% (*n* = 6)	0.547
LACTR	3.85% (*n* = 2)	4% (*n* = 2)	0.9681		3.3% (*n* = 1)	3.3% (*n* = 1)	1
LA Hartmann operation	0% (*n* = 0)	0% (*n* = 0)	1		0% (*n* = 0)	0% (*n* = 0)	1
LAICR	19.23% (*n* = 10)	8% (*n* = 4)	0.0994		15.15% (*n* = 5)	12.12% (*n* = 4)	0.7198
LALAR	3.85% (*n* = 2)	18% (n = 9)	0.0212*		6.06% (*n* = 2)	15.15% (*n* = 5)	0.2304
LALt.hemi.CR	3.85% (*n* = 2)	10% (*n* = 5)	0.2191		6.06% (*n* = 2)	3.3% (*n* = 1)	0.5546
LARt.hemi.CR	26.92% (*n* = 14)	18% (*n* = 9)	0.281		27.27% (*n* = 9)	24.24% (*n* = 8)	0.7783

*P < 0.05

**Table 5:** Operative and postoperative results in QSA and NQS groups before and after PSM

Before PSM	QSA *n* = 52	NQS *n* = 50	*p* value	After PSM	QSA *n* = 33	NQS *n* = 33	*p* value
Operation time (min)	217.48	291.7	0.0002*		224.3	272.24	0.0481*
Blood loss (ml)	22.9	45.54	0.0934		26.55	40.3	0.3838
Conversion	0% (*n* = 0)	8% (*n* = 4)	0.0194*		0% (*n* = 0)	9.09% (*n* = 3)	0.0763
Harvested lymph nodes	20.48	12.42	0.0004*		19.97	13.15	0.0163*
Complications	19.23% (*n* = 10)	28% (*n* = 14)	0.2966		24.24% (*n* = 8)	24.24% (*n* = 8)	1
Clavien–Dindo							
I	7.69% (*n* = 4)	18% (*n* = 9)	0.1186		12.12% (*n* = 4)	18.18% (*n* = 6)	0.4923
II	7.69% (*n* = 4)	6% (*n* = 3)	0.7354		6.06% (*n* = 2)	3.03% (*n* = 1)	0.5546
IIIa	1.92% (*n* = 1)	2% (*n* = 1)	0.9777		3.03% (*n* = 1)	3.03% (*n* = 1)	1
IIIb	1.92% (*n* = 1)	2% (*n* = 1)	0.9777		3.03% (*n* = 1)	0% (*n* = 0)	0.3136
Hospital stay after surgery (day)	12.98	13.78	0.1967		12.7	13.55	0.63
Lymph node dissection level			0.0439*				0.267
D0	5.77% (*n* = 3)	6% (*n* = 3)	0.9605		3.03% (*n* = 1)	6.06% (*n* = 2)	0.5546
D1	3.85% (*n* = 2)	4% (*n* = 2)	0.9681		3.03% (*n* = 1)	3.03% (*n* = 1)	1
D2	9.62% (*n* = 5)	32% (*n* = 16)	0.0052*		12.12% (*n* = 4)	30.3% (*n* = 10)	0.0708
D3	80.77% (*n* = 42)	58% (n = 29)	0.0124*		81.82% (*n* = 27)	60.61% (*n* = 20)	0.057

*P < 0.05

This research has some limitations. We modified using PSM and compared to enhance the research; however, there are still some limitations. It is a retrospective study, and there are certain differences in the patient background. Furthermore, there are more cases in the QS group than in the NQS group, which may result in a difference in results. Moreover, it is thought that there is a difference in the skill level of the caster. ESSQS-QS has considerable laparoscopic surgery experience. However, surgeons performing the procedure in the NQS group may have little experience in laparoscopic surgery. This seems to be a strong factor in the results of our study. Also, our study includes data only from a single facility, and the number of cases is small. A multicenter study with a large sample size is needed to comprehensively evaluate this issue in the future.

## Conclusion

Our results indicate that a laparoscopic colorectal surgery performed by ESSQS-QS leads to good short-term outcomes. ESSQS is potentially useful in maintaining and improving the quality of surgical techniques and in standardization of laparoscopic surgery in Japan.

## Data Availability

All of the data are available without restriction. The data are available from the corresponding author upon reasonable request.

## Abbreviations

ASA: American Society of Anesthesiologists
BMI: Body mass index
*DST*: Double stapling technique
ESSQS: Endoscopic Surgical Skill Qualification System
FEEA: Functional end-to-end anastomosis
JSES: Japan Society for Endoscopic Surgery
LA Hartmann’s operation: Laparoscopy-assisted Hartmann’s operation
LAAPR: Laparoscopy-assisted abdominoperineal resection
LAAR: Laparoscopy-assisted anterior resection
LACAR: Laparoscopy-assisted ascending colon resection
LACDR: Laparoscopy-assisted descending colon resection
LACSR: Laparoscopy-assisted sigmoid colon resection
LACTR: Laparoscopy-assisted transverse colon resection
LALAR: Laparoscopy-assisted low anterior resection
LALt.hemi.CR: Laparoscopy-assisted left hemicolectomy
LARt.hemi.CR: Laparoscopy-assisted right hemicolectomy
PSM: Propensity score matching
QS: Qualified surgeon
QSA: Qualified surgeon assist
